# Supplementary material for: Density and Home Range of Cats in a Small Inhabited Mediterranean Island
Source: Animals (Basel). 2024 Aug 6;14(16):2288. doi: 10.3390/ani14162288 (PMC11350886; doi:10.3390/ani14162288)
Supplement: Supplementary file 1 [file animals-14-02288-s001.zip › animals-3107610-supplementary.pdf]

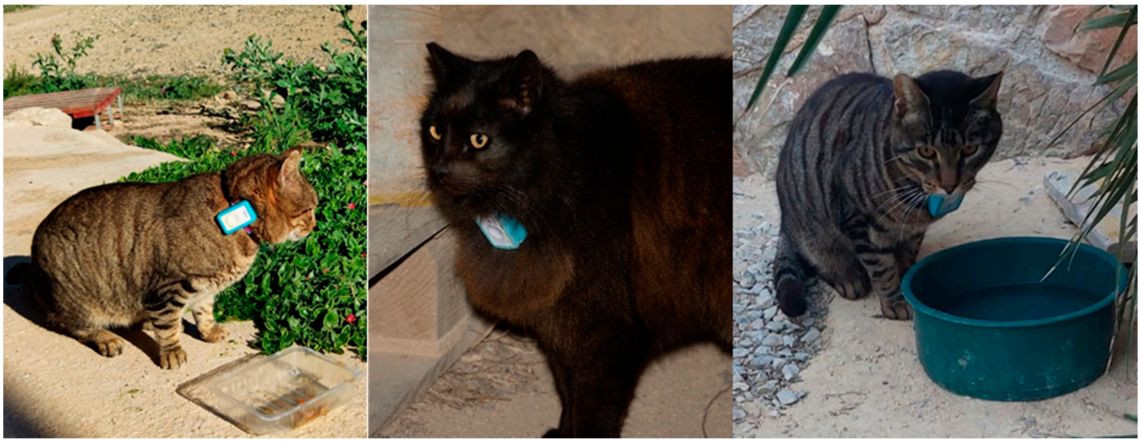

Supplementary Figure S1. Photographs of the 3 cats with the GPS devices attached (from left to right, cats 51, Mía, 28, Negrito and 17, Pascual).

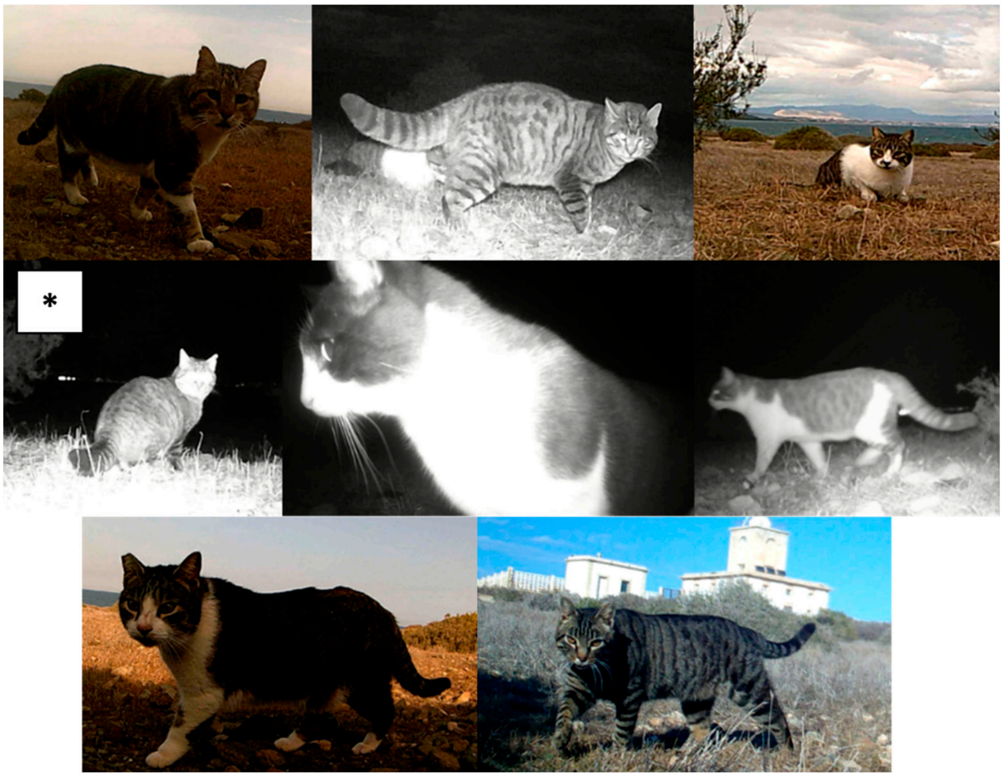

Supplementary Figure S2. Images of the cats from the scrubland area, extracted from the camera trap videos (the only female is identified with an asterisk).
